# Effects of long-term athletic training on muscle morphology and tendon stiffness in preadolescence: association with jump performance

**DOI:** 10.1007/s00421-020-04490-7

**Published:** 2020-09-15

**Authors:** Nikolaos Pentidis, Falk Mersmann, Sebastian Bohm, Erasmia Giannakou, Nickos Aggelousis, Adamantios Arampatzis

**Affiliations:** 1 Department of Training and Movement Sciences, Humboldt-Universität zu Berlin, Berlin, Germany; 2 Berlin School of Movement Science, Humboldt-Universität zu Berlin, Berlin, Germany; 3 Department of Physical Education and Sports Science, Democritus University of Thrace, Komotini, Greece

**Keywords:** Achilles tendon, Muscle hypertrophy, Muscle strength, Plantar flexors, Resistance exercise

## Abstract

**Purpose:**

Evidence on training-induced muscle hypertrophy during preadolescence is limited and inconsistent. Possible associations of muscle strength and tendon stiffness with jumping performance are also not investigated. We investigated the thickness and pennation angle of the gastrocnemius medialis muscle (GM), as indicators for potential muscle hypertrophy in preadolescent athletes. Further, we examined the association of triceps surae muscle–tendon properties with jumping performance.

**Methods:**

Eleven untrained children (9 years) and 21 similar-aged artistic gymnastic athletes participated in the study. Muscle thickness and pennation angle of the GM were measured at rest and muscle strength of the plantar flexors and Achilles tendon stiffness during maximum isometric contractions. Jumping height in squat (SJ) and countermovement jumps (CMJ) was examined using a force plate. We evaluated the influence of normalised muscle strength and tendon stiffness on jumping performance with a linear regression model.

**Results:**

Muscle thickness and pennation angle did not differ significantly between athletes and non-athletes. In athletes, muscle strength was greater by 25% and jumping heights by 36% (SJ) and 43% (CMJ), but Achilles tendon stiffness did not differ between the two groups. The significant predictor for both jump heights was tendon stiffness in athletes and normalised muscle strength for the CMJ height in non-athletes.

**Conclusion:**

Long-term artistic gymnastics training during preadolescence seems to be associated with increased muscle strength and jumping performance but not with training-induced muscle hypertrophy or altered tendon stiffness in the plantar flexors. Athletes benefit more from tendon stiffness and non-athletes more from muscle strength for increased jumping performance.

## Introduction

Regular athletic training loads the musculoskeletal system and initiates adaptation in muscles (Folland and Williams 2007; Andersen and Aagaard 2010), tendons (Arampatzis et al. 2007a; Couppé et al. 2008) and bones (Bennell et al. 1997; Bass et al. 2002). It is well established that muscle size, muscle strength, and tendon stiffness are greater in adolescent athletes compared to untrained controls of similar age (Charcharis et al. 2019; Mersmann et al.^2016^,2017a). Due to substantial changes in muscle-anabolic hormones (Round et al. 1999; Vingren et al. 2010) from early to late adolescence, muscle strength increases rapidly with a similar development in the muscle size (Kanehisa et al. 1995a, b). In preadolescence though, with the basal level of anabolic sex hormones being low (Round et al. 1999; Murray and Clayton 2013) and the endocrine glands likely less responsive to exercise loading (Vingren et al. 2010), the potential effects of exercise on muscle hypertrophy seem limited (Lloyd and Oliver 2012). Several studies reported an increase in muscle strength after various resistance or plyometric training interventions in preadolescent children (Falk and Tenenbaum 1996; Chaouachi et al. 2014a; Cunha et al. 2015; Behm et al. 2017). However, the responsible mechanisms for the observed gains in muscle strength are not well explored, and it is often suggested that training-induced muscle strength gains before puberty be more related to neural adaptations, which include changes in motor unit coordination, rate coding and recruitment, rather than muscle hypertrophy (Faigenbaum et al. 2009b).

It has been shown in some studies (Daly et al. 2004; Sanchis-Moysi et al. 2012, 2017) that long-term athletic training may trigger muscle hypertrophy in preadolescent tennis players. These studies indicate that an increase in muscle size as a consequence of long-term athletic training (at least 2 years, 3–10 h per week) might be an additional mechanism for muscle strength enhancements in children. However, most randomised controlled experiments led to contradictory findings (Ramsay et al. 1990; Granacher et al. 2011) and suggested a negligible effect of resistance exercise on muscle hypertrophy (Granacher et al. 2011). A conceptual difference between these studies was the duration and intensity of the training loading, which might explain the different findings. While the first investigations (Daly et al. 2004; Sanchis-Moysi et al. 2012, 2017) examined athletes who trained systematically for more than 2 years, Ramsay et al. (1990) and Granacher et al. (2011) applied resistance training for 20 and 10 weeks, respectively. Furthermore, the muscle hypertrophy in preadolescent tennis players was observed at the arm (Daly et al. 2004; Sanchis-Moysi et al. 2012) and trunk musculature (Sanchis-Moysi et al. 2017), while both Ramsay et al. (1990) and Granacher et al. (2011) investigated weight-bearing muscles (i.e. knee extensors). Therefore, we can argue that additional investigations examining the effects of systematic resistance training on muscle hypertrophy in preadolescence are needed to better understand musculotendinous adaptive responses in this age. The athletic training in artistic gymnastics is characterised by a high volume of exercises requiring a high level of muscle strength exertion like jumping, landing, and movements on the apparatus in the age before puberty (Pentidis et al. 2019), and is, therefore, appropriate for the investigation of possible training-induced hypertrophy in preadolescence.

Magnetic resonance imaging (MRI) is considered to be the gold standard methodology to measure muscle size due to adequate resolution and good contrast between surrounding tissues, which enable the reliable segmentation of transverse plane images over the full muscle length (Mitsiopoulos et al. 1998). The high expenses and limited accessibility of MRI scanners, however, challenge the assessment of muscle size using MRI. Ultrasonography is an alternative, reliable and feasible methodology to measure muscle morphometrics (i.e. muscle thickness, pennation angle, and fascicle length; Aggeloussis et al. 2010; Giannakou et al. 2011; Marzilger et al. 2018). Several studies found a positive association between muscle morphometric parameters and muscle cross-sectional area (CSA) or muscle volume (Miyatani et al. 2002, 2004; Franchi et al. 2018), establishing muscle morphometrics as reliable predictors for muscle size.

Muscle strength and tendon stiffness affect performance in both daily life activities such as walking (Karamanidis and Arampatzis 2007; Lai et al. 2015) and stair negotiation (Karamanidis and Arampatzis 2009, 2011) as well as during sport-related activities such as running (Arampatzis et al. 2006; Albracht and Arampatzis 2013), sprinting (Stafilidis and Arampatzis 2007) and jumping (Bojsen-Møller et al. 2005; Nikolaidou et al. 2017; Waugh et al. 2017). Although tendons, as mostly collagenous structures, are not able to generate substantial forces, they can significantly affect the potential of muscles for force and muscle power production due to their elasticity (Roberts et al. 1997; Nikolaidou et al. 2017; Bohm et al. 2018, 2019). A balanced adaptation between muscle strength and tendon stiffness seems to favour the storage and release of strain elastic energy (Lichtwark et al. 2007; Orselli et al. 2017) and facilitates the operating conditions of muscle fascicles with regard to the force–length and force–velocity relationships (Azizi et al. 2008). In adults, greater muscle strength and higher tendon stiffness positively affect jumping performance (Bojsen-Møller et al. 2005; Jakobsen et al. 2012; Marián et al. 2016). In preadolescence, some studies are reporting a relationship between muscle strength and jumping height (Ingle et al. 2006; Faigenbaum et al. 2009a; Johnson et al. 2011), but, to our knowledge, there is no information regarding the association of tendon stiffness to jumping performance in this age period. Taken into consideration that plyometric training seems more effective in preadolescents than in adolescents (Moran et al. 2017), the investigation of associations between muscle–tendon properties and jumping performance might contribute to detect possible specific effects of athletic training in this age.

The purpose of the current study was to investigate the thickness and pennation angles of the gastrocnemius medialis muscle (GM) in athletes and non-athletes as indicators for possible long-term training-induced muscle hypertrophy and remodelling in weight-bearing muscles in preadolescence. A second aim was to examine the association of Achilles tendon stiffness and normalised to body mass muscle strength of the plantar flexors with jumping height. We hypothesised to find evidence of training-induced muscle hypertrophy and remodelling in athletes, with greater GM muscle thickness and pennation angles compared to non-athletes. Further, we expected a positive correlation of both Achilles tendon stiffness and normalised to body mass muscle strength of the plantar flexors with jumping performance.

## Methods

### Participants and experimental design

A statistical power analysis in G*Power (3.1.9.2, HUU, Düsseldorf, Germany) was performed for the necessary sample size. We estimated an effect of *d* = 1.2 of the long-term athletic training on muscle morphology, considering the differences from an earlier study between preadolescent tennis player and untrained participants (*d* = 1.5; Sanchis-Moysi et al. 2012). A lower effect size was selected due to a minor difference in training history between studies, and the lower potential for muscle hypertrophy of the weight-bearing muscles (Abe et al. 2000). For a power of 0.8 and allocation ratio of 1.3 (expecting a smaller sample of untrained children), a sample size of 11 participants per group would be sufficient to achieve the desired statistical power of the expected outcome. Therefore, eleven untrained preadolescent participants (6 females; ∼ 3 h of sports activity per week in school and no participation in systematic training; hereafter referred to as non-athletes) and 21 similar-aged athletes of artistic gymnastics (15 females; ∼ 20 h of training per week) participated in the present study (Table 1). The pubertal status of the participants was assessed by their parents, by determining the Tanner stage according to drawings of the respective features (Marshall and Tanner 1969, 1970). At the time of data acquisition, the training intensity and volume of the athletes had been documented for the last 6 months and averaged to ∼ 5 h of specific muscle strength and jumping training and ∼ 12 h of main on-apparatus gymnastic training per week, including plyometric and landing exercises (for a detailed description of the training see Pentidis et al. 2019).

**Table 1:** Anthropometric data for preadolescent athletes and non-athletes

	Athletes	Non-athletes	Cohen’s *d*
Age (years)	9.2 ± 1.7	9.0 ± 1.7	0.70
Height (cm)	131.1 ± 8.0	134.6 ± 11.7	0.06
Body mass (kg)	28.9 ± 6.4	31.1 ± 9.0	0.09
BMI (kg/m^2^)	16.6 ± 2.0	16.8 ± 3.0	0.02

Values are means ± SD

*BMI* body mass index

No statistically significant differences (*p* > 0.05) were found between the two groups

The study was approved by the Ethics Committee of the Democritus University of Thrace (approval number B 2235) and all participants (and respective legal guardians) gave written informed consent in accordance with the Declaration of Helsinki. The measurements of plantar flexor muscle strength, GM muscle morphology and Achilles tendon stiffness were carried out on the right leg, following a standardised warm-up including 3 min of running, ten submaximal jumps and five submaximal isometric plantar flexion contractions. Four females (two of each group) had a dominant left leg (i.e. leading leg in a forward fall). However, the results from Bohm et al. (2015) suggest that in persons, who do not engage in physical activity related to strong unilateral loading, no significant differences in muscle strength or tendon stiffness between sides are to be expected.

### Measurement of maximum ankle joint moment

**Fig. 1:**
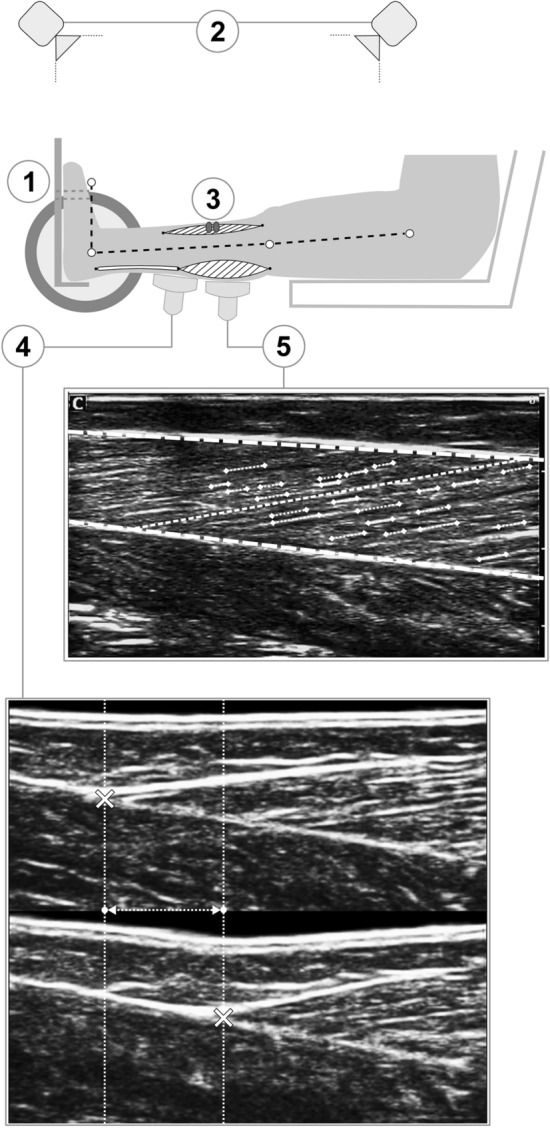
Schematic representation of the experimental setup. View on the medial part of the right leg. A dynamometer (**1**) was used to measure ankle joint moments, while kinematic recordings (**2**) were used for inverse dynamics and electromyography (**3**) for the consideration of antagonist coactivation. Ultrasound imaging was used to assess Achilles tendon elongation (**4**; the crosses represent the gastrocnemius medialis myotendinous junction and the vertical lines illustrate their displacement during the contraction) and gastrocnemius medialis architecture (**5**; the digitalisation of the aponeuroses and the fascicle portions, indicated by the thick dashed line and pointed lines, respectively, and the calculated reference fascicle, represented by the thin dashed line, are overlaid over the ultrasound image)

For the evaluation of plantar flexor muscle strength, the participants performed five isometric maximum voluntary plantar flexion contractions (MVC) on a dynamometer (Cybex 6000, Ronkonkoma, NY, USA) at 0°, 5°, 10°, 15°, and 20° of dorsiflexion (tibia perpendicular to the sole = 0°, ankle angle determined via a manual goniometer) (Fig. 1). The hip angle was set to 70° (supine = 0°) and the knee was fully extended (0°). The resultant ankle joint moments were calculated using inverse dynamics (Arampatzis et al. 2005a) to consider the effects of the misalignment between the ankle joint and dynamometer axes as well as the effects of gravitational forces on the measured moments. For this reason, kinematic data were recorded using a Vicon motion capture system (version 1.8.5; Vicon Motion Systems, Oxford, United Kingdom) integrating eight cameras operating at 100 Hz. Seven reflective markers were fixed to the following anatomical landmarks: trochanter major, lateral and medial femoral epicondyles, lateral and medial malleoli, the dorsal gap between the distal metaphysis of the second and third metatarsals and calcaneus. To consider the gravitational forces of the dynamometer-footplate and foot, the ankle was passively rotated by the dynamometer at 5°/s in an additional trial. The contribution of the antagonistic moment during the maximum isometric plantar flexion contractions was calculated based on the relationship of the electromyographic (EMG) activity of tibialis anterior and the exerted moment during submaximal dorsiflexion contractions as reported by Mademli et al. (2004). The EMG activity of the tibialis anterior muscle during the MVCs was captured at 1000 Hz (Myon m320RX; Myon, Baar, Switzerland) and transmitted to the Vicon system via an A/D converter. In summary, the maximum ankle joint moments reported in this study are adjusted for the effects of gravitational forces, the effects of the ankle joint axis misalignment to the dynamometer axis and the effects of the antagonistic moment.

### Measurement of gastrocnemius medialis muscle morphology

GM muscle thickness and pennation angle were measured at rest, at an ankle joint angle of 0°. A 6-cm linear array ultrasound probe (7.5 MHz; Chison, Model Q3, Wuxi, China) was placed over the belly of the inactive muscle along its longitudinal axis at ∼ 50% of its length. The ultrasound images were analysed offline in a custom-written MATLAB interface (version R2012b; MathWorks, Natick, MA, USA). The upper and deeper aponeuroses were defined by tracking four reference points along both aponeuroses and applying a linear least-squares fitting (Marzilger et al. 2018). The visible features of multiple fascicles (on average 20 ± 4) were then digitised and a reference fascicle based on the average inclination of the fascicle portions and the distance of the aponeuroses (Fig. 1) was calculated. Average values of ten frames were used for further analysis to determine the length of the reference fascicle (fascicle length), the angle between the reference fascicle and the deeper aponeurosis (pennation angle), and the distance between the upper and deeper aponeuroses (muscle thickness). The average value of five trials was used for data analysis.

### Assessment of Achilles tendon stiffness

For establishing the force–elongation relationship of the Achilles tendon, the same ultrasound probe used for the muscle morphology measurements was fixed overlying the myotendinous junction (MTJ) of the GM. The displacement of the MTJ was captured at 85 Hz during five trials of isometric ramp contractions (i.e., increasing effort steadily from rest to maximum in ∼ 4 s). To avoid fatigue, the MVCs were performed with at least 3-min rest between trials. To take into consideration the effect of the inevitable joint angular rotation on the displacement of the MTJ during ramp contractions, an additional trial with a passive rotation of the ankle joint at 10°/s was captured to determine the corresponding displacement of the MTJ as a function of joint angle (Arampatzis et al. 2005b). The difference between ramp contractions and the corresponding passive joint rotation of the MTJ displacement was defined as the tendon elongation. The resting length of the Achilles tendon was defined as the length of the curved path from calcaneal tuberosity (insertion of the Achilles tendon) to the MTJ with the ankle at 20° of plantar flexion (De Monte et al. 2006). The tendon excursion approach was used to calculate the tendon moment arm (Fath et al. 2010). Briefly, in a range of negligible passive tendon strain (i.e., 5° dorsiflexion and − 10° plantar flexion; De Monte et al. 2006), the relationship of the MTJ displacement and ankle joint angle changes, was used to assess the Achilles tendon moment arm. The moment arm was adjusted for changes from rest to maximum isometric contraction using the data from Maganaris et al. (2000). The tendon force was then calculated by dividing the ankle joint moment by the tendon moment arm. The displacement of the MTJ was tracked manually frame-by-frame by one experienced observer (N.P.) using a custom-written MATLAB interface. For achieving excellent reliability (Schulze et al. 2012), averaged values of the five ramp contractions were used for the force–elongation relationship. The resultant force–elongation curve was fitted using a second-order polynomial and then tendon stiffness was calculated as a slope of a linear regression between 50 and 100% of the peak tendon force. To synchronise the ultrasound images with the data captured in the Vicon system, an externally induced voltage peak (of 3 V) was used.

### Measurement of jump performance

The participants performed three countermovement jumps (CMJ) and three squat jumps (SJ) with maximum effort. For the CMJ, the participants were instructed to quickly squat from the standing position to a knee angle of approximately 90° (checked by visual observation) and to jump immediately afterwards. For the SJ, they were instructed to hold the starting position with a knee angle of 90° for 3 s and to perform the jump without countermovement. In both jumps, the instructions were to jump as high as possible with their arms akimbo. During each jump, a force plate sampling at 1000 Hz (Kistler 9281CA, Winterthur, Switzerland) was used to measure the vertical ground reaction force (GRF) and the vertical take-off velocity of the centre of mass was calculated by integrating the vertical GRF over time. The trial with the greatest jump height was considered in the statistical analysis.

### Statistics

The statistical analyses were conducted in SPSS (version 25.0; IBM, Armonk, NY, USA). Normality of the data was checked using the Shapiro–Wilk test. For the normally distributed parameters, a one-way analysis of covariance (ANCOVA) was performed to investigate differences between groups (i.e. athletes and non-athletes), with the Tanner scale as a covariate. Furthermore, the effect size Cohen’s *d* was calculated based on the partial eta squared. Body mass and body mass index were not normally distributed and, therefore, the non-parametric Mann–Whitney *U* test for independent samples was used. The effect sizes from the non-normally distributed parameters were transformed to Cohen’s *d* to be comparable with the normally distributed parameters. Effect sizes will be referred to as small (0.2 ≤ *d* < 0.5), medium (0.5 ≤ *d* < 0.8), and large (*d* ≥ 0.8) (Cohen 1988). To examine relationships between parameters, the Pearson correlation coefficient was used. The level of significance for all investigations was set to *α* = 0.05.

To evaluate the influence of Achilles tendon stiffness and normalised muscle strength of the plantar flexors on jump performance for both athletes and non-athletes, a linear regression model (equation see below) was applied. Achilles tendon stiffness (${K}_{\mathrm{AT}})$ and normalised ankle joint moment (${M}_{\mathrm{norm}})$ of each participant were used as predictors for the corresponding jump height of the squat or countermovement jump. The model was formulated in a way that each group had its own intercept and slope coefficients (${a}_{i},{b}_{i}, {c}_{i}$ for $i\in \{\mathrm{1,2}\}$) to be able to compare the influence of both predictors.

The equation was,

$$h = (a_{1} + b_{1} K_{{{\text{AT}}}} + c_{1} M_{{{\text{norm}}}} )G_{A} + (a_{2} + b_{2} K_{{{\text{AT}}}} + c_{2} M_{{{\text{norm}}}} )G_{{{\text{NA}}}} + \varepsilon_{j,}$$

where the group indicators ${G}_{\mathrm{A}}$ (athletes) and ${G}_{\mathrm{NA}}$ (non-athletes) were one for the respective group, zero otherwise. The residuals for each participant are noted as ${\varepsilon }_{j}$ ($j=1,\dots , n)$. To compare the influence of the predictors (i.e. Achilles tendon stiffness and normalised ankle joint moment) on jump performance for the different groups the standardised coefficients (*β*) of the regression analysis were used.

## Results

**Fig. 2:**
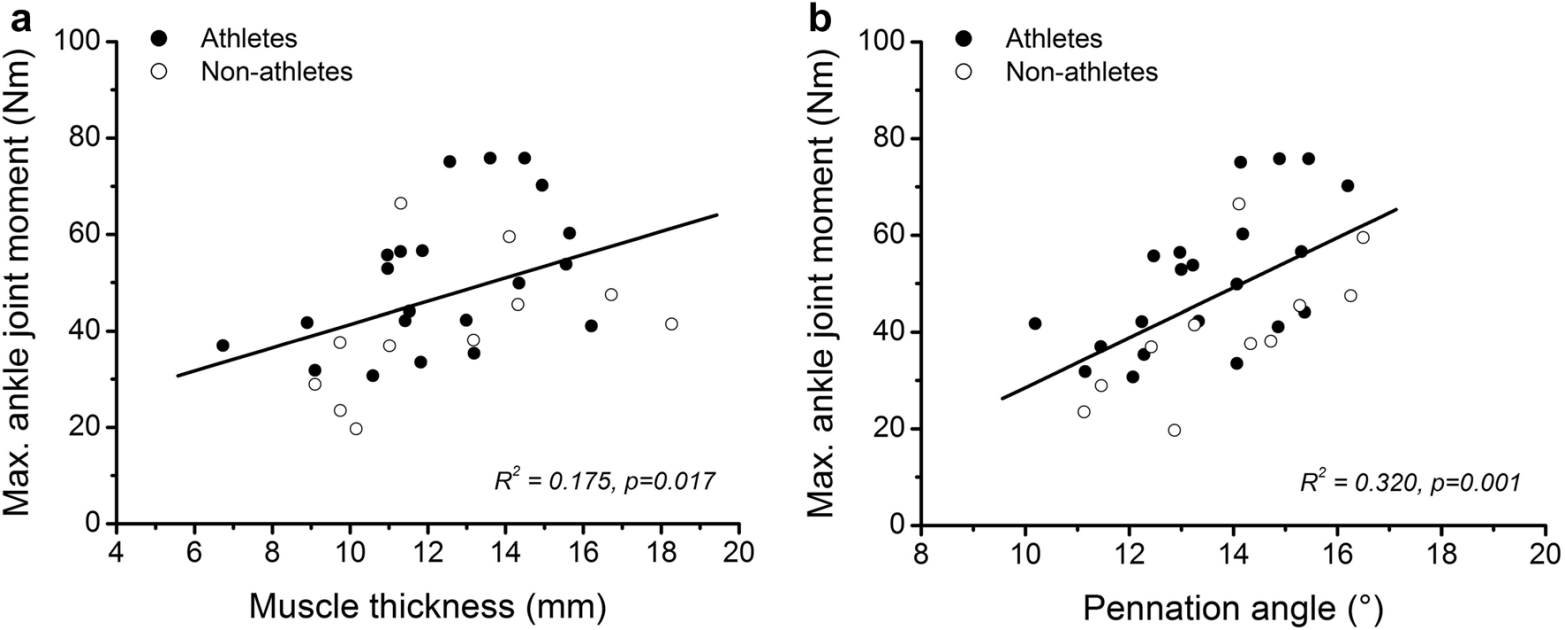
Relationship of **a** muscle thickness of the gastrocnemius medialis and **b** pennation angle of gastrocnemius medialis with the maximum ankle joint moment in preadolescent athletes and non-athletes

There were no significant differences in age (*d* = 0.70, *p* = 0.069), height (*d* = 0.06, *p* = 0.859), body mass (*d* = 0.09, *p* = 0.815) and body mass index (*d* = 0.02, *p* = 0.984) between the two groups (Τable 1). Fourteen athletes (66.6%) were categorised to Tanner Stage I and seven (33.4%) to Tanner Stage II. In the non-athletes group, five participants (45%) were in Tanner Stage I and six (55%) in Tanner Stage II. The athletes showed significantly higher jumping height in both SJ (*d* = 1.91, *p* < 0.001) and CMJ (*d* = 2.24, *p* < 0.001; Table 2) compared to the non-athletes. The CMJ height was 1.87 cm and 0.53 cm greater than in SJ in athletes and non-athletes, respectively, but without any statistically significant differences between the two groups (*d* = 0.543, *p* = 0.155). Both maximum and normalised ankle joint moments were significantly greater in athletes (*d* = 1.33, *p* = 0.001; *d* = 1.4, *p* = 0.001, respectively; Table 2), but the Achilles tendon stiffness did not differ significantly between the two groups (*d* = 0.36, *p* = 0.341; Table 2). There were no significant differences between the two groups in pennation angle of the GM (*d* = 0.06, *p* = 0.895) and muscle thickness (*d* = 0.11, *p* = 0.764; Table 2). The maximum ankle joint moment was significantly correlated with the muscle thickness (*r* = 0.419, *p* = 0.017) and pennation angle (*r* = 0.565, p = 0.001; Fig. 2).

**Table 2:** Jumping height, maximum and normalised ankle joint moments, Achilles tendon stiffness and morphometrics of the gastrocnemius medialis muscle of the preadolescent athletes and non-athletes

	Athletes	Non-athletes	Cohen’s *d*
SJ height (cm)*	21.1 ± 3.5	15.5 ± 2.0	1.91
CMJ height (cm)*	22.9 ± 3.8	16.0 ± 3.8	2.24
Maximum ankle joint moment (Nm)*	50.6 ± 14.6	40.5 ± 14.1	1.33
Normalised ankle joint moment (Nm/kg)*	1.74 ± 0.32	1.31 ± 0.33	1.4
Achilles tendon stiffness (N/mm)	117.2 ± 33.6	106.4 ± 32.7	0.36
Pennation angle (°)	13.5 ± 1.6	13.8 ± 1.8	0.06
Muscle thickness (mm)	12.3 ± 2.4	12.5 ± 3.0	0.11

Values are means ± SD. Ankle joint moment normalised to body mass

*SJ* squat jump, *CMJ* countermovement jump

*Significant difference between athletes and non-athletes, *p* < 0.05

**Fig. 3:**
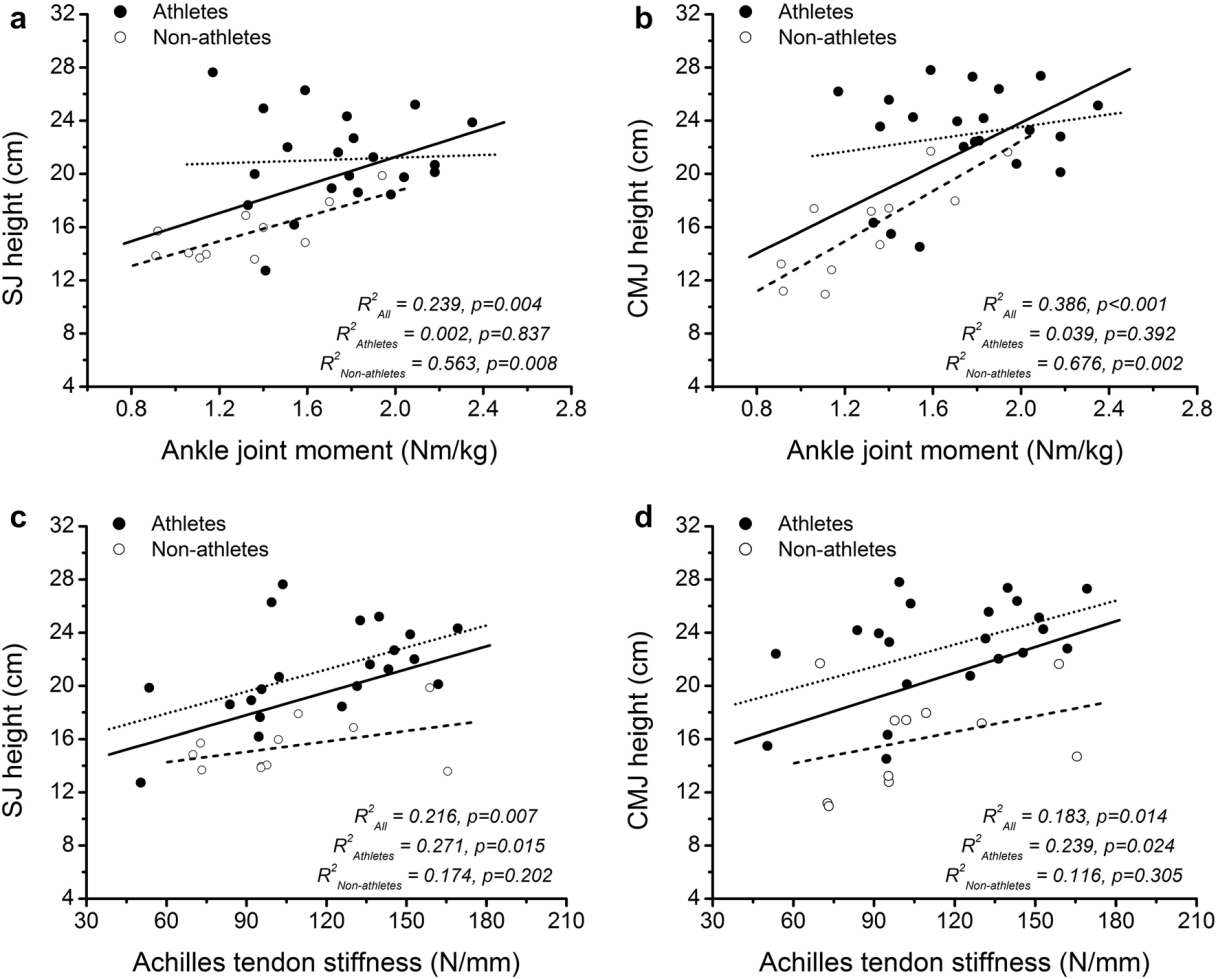
Relationship of **a** squat jump (SJ) height and **b** countermovement jump (CMJ) height with normalised to body mass ankle joint moment, and **c** squat jump height and **d** countermovement jump height with Achilles tendon stiffness in athletes and non–athletes

Including all participants in the regression analysis, there was a significant positive correlation between the normalised ankle joint moment and jumping height in both SJ (*r* = 0.489, *p* = 0.004) and CMJ (*r* = 0.622, *p* < 0.001; Fig. 3). Achilles tendon stiffness also was significantly associated to jumping height (SJ: *r* = 0.465, *p* = 0.007; CMJ: *r* = 0.428, *p* = 0.014; Fig. 3). There was a significant relationship between Achilles tendon stiffness and jumping height (SJ: *r* = 0.521, *p* = 0.015; CMJ: *r* = 0.489, *p* = 0.024) in athletes, but no significant association in non-athletes (SJ: *r* = 0.417, *p* = 0.202; CMJ: *r* = 0.341, *p* = 0.305). The normalised maximum ankle joint moment related significantly to jumping height in non-athletes (SJ: *r* = 0.750, *p* = 0.008; CMJ: *r* = 0.822, *p* = 0.002) and did not show any significant relationship in athletes (SJ: *r* = 0.045, *p* = 0.837; CMJ: *r* = 0.197, *p* = 0.392). The standardised coefficients of the linear regression model showed that Achilles tendon stiffness was the significant predictor for both the SJ and CMJ jumping heights in the athletes (Table 3). In the non-athletes, the normalised ankle joint moment was the significant predictor for the CMJ jumping height, while in SJ neither ankle joint moment nor tendon stiffness was a significant predictor for the jumping height (Table 3).

**Table 3:** Standardised coefficients of the linear regression model for predicting the jump height of preadolescent athletes and non-athletes with the normalised ankle joint moment and Achilles tendon stiffness as predictors

		Athletes		Non-athletes	
	Predictor	Standardised coefficients (*β*)	Level of significance	Standardised coefficients (*β*)	Level of significance
SJ height	Norm. ankle joint moment	− 0.105	*p* = 0.490	0.182	*p* = 0.157
	Predictor				
	Achilles tendon stiffness	0.299	*p* = 0.005*	0.010	*p* = 0.926
	Norm. ankle joint moment				
CMJ height		0.039	*p* = 0.809	0.376	*p* = 0.009*
	Achilles tendon stiffness	0.249	*p* = 0.024*	− 0.038	*p* = 0.734

Ankle joint moment normalised to body mass

*SJ* squat jump, *CMJ* countermovement jump

*Significant predictor for the jumping height, *p* < 0.05

## Discussion

In this study, we hypothesised to find greater muscle thickness and pennation angle in athletes and an association between muscle–tendon properties and jumping performance. No significant differences in muscle morphometrics between the two groups were found, but normalised muscle strength, as well as Achilles tendon stiffness, were associated with jumping height, indicating the importance of the triceps surae muscle–tendon properties on jumping performance in preadolescent children. Therefore, the results only partly confirmed our hypotheses.

It is well documented by numerous studies, reviews and meta-analyses that preadolescents have the capacity to increase their muscle strength through physical resistance exercise (Malina 2006; Behringer et al. 2010; Chaouachi et al. 2014a; Granacher et al. 2016, 2018). There are studies demonstrating that after 6–8 weeks of resistance training, muscle strength increases by 30–40% (Hassan 1991; Faigenbaum et al. 1999). Also, plyometric training for 8 weeks can lead to an increase in muscle strength of 25% (Chaouachi et al. 2014b). The athletes who participated in the current study trained for 20 h/week with various plyometric and strength exercises. The significantly greater plantar flexor maximum and normalised muscle strength of 25% and 33%, respectively, that we found in athletes compared to non-athletes, can be attributed to this amount of training. Furthermore, we found a significant relationship between GM thickness and pennation angle with the maximum ankle joint moment. As muscle thickness is a predictor of muscle size and the angle of pennation an important modulator of the physiological cross-sectional area of a muscle (Aagaard et al. 2001; Giles et al. 2015), these findings provide evidence that plantar flexor strength is strongly dependent on muscle morphometrics and size in preadolescent children. However, both muscle thickness and angle of pennation did not differ significantly between athletes and non-athletes, indicating no training-induced hypertrophy in the investigated gymnasts.

Recently, cross-sectional studies (Daly et al. 2004; Sanchis-Moysi et al. 2012, 2017) that investigated trunk and upper extremity muscles provide evidence that long-term exercise loading induces muscle hypertrophy in preadolescent athletes. Sanchis-Moysi et al. (2012) investigated preadolescent tennis players (11 years, Tanner scale 1–2) with a training history and frequency comparable to the present study’s athletes and found significantly greater muscle volume in the dominant compared to the contralateral arm (13%) and in comparison with an age-matched control group of non-athletes (16%), indicating training-induced hypertrophy in preadolescent tennis players. Even though this study’s athletes of artistic gymnastics trained on average for 4 years, 5–6 trainings per week and ~ 3.5 h per training, the results showed that even with this training history, which involved high-intensity loading, no indications for muscle hypertrophy occurred. The greater potential for muscle hypertrophy of the upper body (non-weight bearing) than the lower body (weight-bearing) muscles (Abe et al. 2000) due to long-term exercise loading may explain the different results between the aforementioned studies (Daly et al. 2004; Sanchis-Moysi et al. 2012, 2017) and the present study. Our results are in line with intervention studies on the lower extremity muscles (Vrijens 1978; Ramsay et al. 1990; Granacher et al. 2011), which did not find any significant alteration in muscle-CSA after 8–20 weeks of resistance training. Muscle strength adaptations in preadolescence are more likely to occur at the neuromuscular level (Granacher et al. 2011) and training-induced changes in muscle size, at least in weight-bearing muscles, seem not to play a crucial role. There are reports that preadolescent children show a tendency towards a lower percentage of fast-twitch fibres compared to adults (Oertel 1988; Dotan et al. 2012; Verdijk et al. 2014), which have been attributed a greater hypertrophic response to resistance training compared to slow-twitch fibres (Hortobágyi et al. 1996; Andersen and Aagaard 2000; Aagaard et al. 2001). Therefore, one might suggest that the lack of hypertrophy in our athletes could be related to a low percentage of fast-twitch fibres. However, the findings are inconsistent and other studies are reporting similar percentage of fast and slow-twitch fibres in children and adults (Bell et al. 1980; Vogler and Bove 1985; Metaxas et al. 2014) and similar training-induced hypertrophy in slow and fast-twitch fibres (Mero et al. 2013; Bogdanis et al. 2018). Therefore, neither the literature reports nor the experimental design of the current study allows for a clear valuation of this possibility*.*

It is well established that individuals with higher maximum muscle strength commonly also feature higher tendon stiffness to be able to tolerate the mechanical loading placing upon the tendon by the working muscle (Arampatzis et al. 2007a; Charcharis et al. 2019; Seynnes et al. 2009). Even though our data indicate a positive association between maximum ankle joint moment and Achilles tendon stiffness (*r* = 0.472, *p* = 0.006), Achilles tendon stiffness did not differ significantly between athletes and non-athletes. Jumping, landing and plyometric exercises prevailed during the training in the investigated athletes. Plyometric training is not the most appropriate training stimulus for tendon adaptation (Bohm et al. 2019; Kubo et al. 2017; Mersmann et al. 2017b) and does not lead to major adaptive alterations in tendon mechanical properties (Burgess et al. 2007; Fouré et al. 2009, 2011; Bohm et al. 2014). In our earlier studies (Arampatzis et al. 2010, 2007b; Bohm et al. 2014)^,^ we found that cyclic loading of the tendon with strain values between 4.5 and 6.5% and with a frequency of 0.17 Hz (3-s loading and 3-s relaxation per repetition) provides the most advantageous adaptive responses of tendon mechanical and morphological properties in vivo. This loading structure was not provided by the sport-specific training in the athletes of the present study. Though both muscle strength and tendon stiffness can be enhanced in prepubescent children with appropriate training (Waugh et al. 2014), muscle and tendon responses to exercise loading in preadolescence may still be limited due to the lower concentration of sex hormones compared to adolescence (Round et al. 1999; Veldhuis et al. 2000). The concentration of sex hormones affects muscle and tendon protein metabolism and promote muscle–tendon adaptive responses (Doessing et al. 2010; Vingren et al. 2010; Hansen and Kjaer 2014). The Tanner Stage I and II of the investigated athletes, where the level of sex hormones is negligible (Round et al. 1999), may explain the absence of marked differences in Achilles tendon stiffness and muscle morphometrics between the two groups.

The increase in jump height from SJ to CMJ was not significantly different between athletes and non-athletes and in average 7.3%, which is in line with the differences reported in adults (Anderson and Pandy 1993; Bobbert et al. 1996; Nikolaidou et al. 2017). It is well documented that the higher activation of the lower extremity muscles during the propulsion phase and especially at the beginning of the push-off is the responsible mechanism for the higher jumping height in CMJ (Bobbert and Casius 2005; Nikolaidou et al. 2017). Therefore, the differences in muscle strength between groups may affect general jump height but not the differences between the two types of jump. We found significant relationships of normalised to body mass ankle joint moment and Achilles tendon stiffness to jumping height, indicating that both muscle strength of the plantar flexors and Achilles tendon stiffness influence jumping performance in preadolescent children. The positive effect of muscle strength on jumping performance is well documented in both adults and preadolescents (Stone et al. 2003; Faigenbaum et al. 2009b; Johnson et al. 2011; Smilios et al. 2013; Marián et al. 2016). Reports about the effect of tendon stiffness on jumping performance are scarce and limited to adults (Bojsen-Møller et al. 2005; Burgess et al. 2007; Kubo et al. 2007). Our study provides novel information concerning the relevance of Achilles tendon stiffness on jumping height in preadolescence and supports the important contribution of tendon mechanical properties for performance in sports (Arampatzis et al. 2006; Fletcher et al. 2010). The jumping height in both SJ and CMJ was on average 40% higher in athletes compared to non-athletes. Further, athletes seemed to benefit more from Achilles tendon properties than non-athletes. Our regression model shows that the determining factor for the jumping height in athletes was the Achilles tendon stiffness and for the non-athletes the normalised muscle strength.

During the execution of SJ and CMJ, there is a relevant interaction between the contractile and elastic elements of the plantar flexors, which affect both the energy storage and release of the Achilles tendon as well as the muscular work and power production (Kurokawa et al. 2001, 2003). The contractile element in both jumps produce mechanical work mainly in the first part of the propulsion phase and save work as strain energy in the tendon (Kurokawa et al. 2001, 2003). In the second part of the propulsion, the tendon strain energy is returned and the contractile element contracts almost isometrically, thus without any relevant energy gain (Kurokawa et al. 2001, 2003). These reports evidence a fine-tuning between muscle and tendon for high jumping performance, which might be improved in athletes due to the specific modalities of the gymnastic training. The advantages of plyometric training to improve jumping height are well known in adults (Burgess et al. 2007; Kubo et al. 2017) and preadolescents (Kotzamanidis 2006; Meylan and Malatesta 2009; Johnson et al. 2011). The specific gymnastics training of the investigated athletes, which included a variety of jumping and landing exercises, may have improved the interaction between muscle and tendon, allowing athletes to use tendon elasticity more effectively and enhance jumping performance. A recent meta-analysis (Moran et al. 2017) provides evidence that plyometric training during preadolescence is more beneficial for developing jumping performance compared to adolescence, suggesting a higher potential for improvements of muscle–tendon interaction in preadolescence.

The reliability of muscle strength measurements in children has been reported as moderate (Fagher et al. 2016) to high (Kellis et al. 1999; Moreau et al. 2008). Recently, a meta-analysis study (Muñoz-Bermejo et al. 2019) reported high reliability (intraclass correlations coefficient (ICC) from 0.84 to 0.90) of muscle strength measurements in children and concluded that isokinetic dynamometry is a valid method for muscle strength assessment in this age. We used a valid and robust methodology for the muscle strength assessment where the moments measured at the dynamometer have been corrected for the axis misalignment and gravitational forces (Arampatzis et al. 2005a). We also considered the contribution of the antagonist tibialis anterior in the ankle joint moment during the MVCs (Mademli et al. 2004). In the present study, the ICC of the peak forces during the five ramp contractions was 0.952, demonstrating that children were able to reproduce the maximum ankle joint moment. The measurements of the muscle architecture show high intraday (ICCs higher than 0.94) and interday (ICCs 0.60–0.96) reliability (Marzilger et al. 2018). For the assessment of the Achilles tendon stiffness, we used the average values of five ramp contractions to achieve high reliability (> 0.95, Schulze et al. 2012). We are confident that the used methodologies are well established and contribute to accurate and reliable muscle strength, muscle architecture and tendon stiffness measurements. In our study, the percentage of females included was slightly higher in athletes (71%) compared to non-athletes (55%). However, 2–3 years before the time of peak height velocity, which was the age of the investigated children, the levels of insulin-like growth factor 1 (IGF-1), testosterone and estradiol show quite similar values between females and males (Round et al. 1999; Veldhuis et al. 2000). Furthermore, the cross-sectional area of the lower extremity muscles do not seem to differ between females and males (Kanehisa et al. 1994, 1995b), indicating similar muscle growth and, therefore, this slightly different distribution is unlikely to significantly affect the findings.

In conclusion, the present study provides evidence that athletic training in preadolescence can increase muscle strength and jumping height, probably by superior neuromuscular coordination, without significant training-induced muscle hypertrophy or alterations of Achilles tendon properties. The relationships between normalised ankle joint moment, Achilles tendon stiffness and jumping height highlight the significant contribution of both muscle strength of the plantar flexors and Achilles tendon stiffness to jumping performance at this age. Furthermore, due to the specific training modalities of the investigated artistic gymnasts, which can improve the interaction between muscle and tendon by advantageous neuromuscular coordination, the athletes demonstrated a greater benefit from high levels of Achilles tendon stiffness to augment jumping performance. These results suggest that the interaction between muscle and tendon plays a significant role in jumping performance in preadolescence and particularly in preadolescent athletes.

## Abbreviations

ANCOVA: Analysis of covariance
CMJ: Countermovement jump
CSA: Cross-sectional area
EMG: Surface electromyography
GM: Gastrocnemius medialis muscle
GRF: Ground reaction force
ICC: Intraclass correlation coefficient
MRI: Magnetic resonance imaging
MTJ: Myotendinous junction
MVC: Maximal voluntary isometric contraction
SJ: Squat jump
